# Systems and precision medicine approaches to diabetes heterogeneity: a Big Data perspective

**DOI:** 10.1186/s40169-017-0155-4

**Published:** 2017-07-25

**Authors:** Enrico Capobianco

**Affiliations:** Center for Computational Science, University of Miami, Miami, FL USA

**Keywords:** Diabetes, Systems and precision medicine, Electronic health records

## Abstract

Big Data, and in particular Electronic Health Records, provide the medical community with a great opportunity to analyze multiple pathological conditions at an unprecedented depth for many complex diseases, including diabetes. How can we infer on diabetes from large heterogeneous datasets? A possible solution is provided by invoking next-generation computational methods and data analytics tools within systems medicine approaches. By deciphering the multi-faceted complexity of biological systems, the potential of emerging diagnostic tools and therapeutic functions can be ultimately revealed. In diabetes, a multidimensional approach to data analysis is needed to better understand the disease conditions, trajectories and the associated comorbidities. Elucidation of multidimensionality comes from the analysis of factors such as disease phenotypes, marker types, and biological motifs while seeking to make use of multiple levels of information including genetics, omics, clinical data, and environmental and lifestyle factors. Examining the synergy between multiple dimensions represents a challenge. In such regard, the role of Big Data fuels the rise of Precision Medicine by allowing an increasing number of descriptions to be captured from individuals. Thus, data curations and analyses should be designed to deliver highly accurate predicted risk profiles and treatment recommendations. It is important to establish linkages between systems and precision medicine in order to translate their principles into clinical practice. Equivalently, to realize their full potential, the involved multiple dimensions must be able to process information ensuring inter-exchange, reducing ambiguities and redundancies, and ultimately improving health care solutions by introducing clinical decision support systems focused on reclassified phenotypes (or digital biomarkers) and community-driven patient stratifications.

## Introduction

### Big Data in biomedicine

Currently, being able to perform analytics over Big Data represents the real challenge for many types of organizations, including medical ones. By establishing new forms of collaborative research, next generation analytics is emerging and fueling solutions with a clear impact on clinical decision-making processes. This process responds to one of the main goals of personalized medicine: advancing knowledge of complex diseases. Considering for instance diabetes, this means to rely on the prediction of risks of disease development and also on the control of the disease progression to avoid further well-known complications. The complexity of such tasks is exemplified by the role of associated disorders and comorbidities, and it is further augmented by other instruments that are gaining relevance, especially genomics and electronic health records (EHRs) [1]. Eventually, their integration should improve the clinical decision-making process.

### Emerging trends in diabetes research

High-genomic content EHRs could be extremely useful to cover current knowledge gaps in diabetes data analyses [2]. Type-2 diabetes (T2D) suggests that a major role is played by both environmental and genetic factors. While genetic loci contribute only to a certain extent to heritability, GWAS and EWAS have identified only up to certain limited extent Due to the multiple data sources that are currently examined, epidemiological and clinical studies may try to explain T2D by gene-environment interactions or epigenetic changes regulating gene expression levels. For instance, some of known markers considered important for treatment purposes, lead to stress the role for subclinical inflammatory events (secretion of TNF and pro-inflammatory cytokines) that might be seen as early signals. Other regulatory mechanisms are associated to transcription factors, say NFkB, circulating microRNAs, and epigenetic modifications affecting gene expression (histone hyperacetylation and chromatin remodeling) [3]. Novel biomarker discovery will also benefit from comprehensive screenings generated by next generation sequencing (NGS).

### Epigenetics

Apart from genetic patterns, environmental factors are very informative for diabetes. Epigenetics is acquiring relevance particularly from NGS applications [4]. In particular, a strong impact is expected from non-coding RNAs (ncRNAs), whose identification is opening a new frontier in the study of human pathophysiology. As ncRNAs are known to play a regulatory role in many developmental contexts, a variety of catalogues of lncRNAs has been made available from various studies. Recently, RNA-Seq experiments have pinpointed 1734 protein coding genes and 175 lncRNAs significantly up/down regulated on adipocytes [5, 6]. The enrichment obtained from annotations and control exerted by known transcription factors (Ppary and Cepbalpha) showed relevance for hallmarks of adipogenesis. Notably, a transcriptome map of human pancreatic islets and Beta-cells has revealed about 1100 intergenic and antisense islet-cell lncRNAs [7]. A few examples of dysregulations occurred in T2D or were shown to map to genetic loci underlying diabetes susceptibility. Most of them run antisense from the coding gene, and it is relevant that they have been shown to control the transcription of protein coding genes in *cis* modality.

Understanding T2D molecular etiology also depends on the abnormal Beta cell function or growth. Islet cell dysfunction is central to T2D pathophysiology. In particular KCNQ1QT1 and HI-LNC45 are lncRNAs significantly differential between T2D islets compared to non-diabetic ones. As for the genetic susceptibility for diabetes, ABCC8/KCNJ11 is associated with neonatal diabetes, GATA6 is associated with pancreas agenesis, and HNF1A is associated with monogenic diabetes. Other unique features of beta-cells transcriptome (with over 1000 lincRNAs expressed in mouse) were obtained from another study [8].

### Diabetes and cancer

Two recent studies have examined linkages between diabetes and cancer, two diseases with increasing prevalence and mutual dependence [9–12]. The first study considered cancer risk among T2D patients [13]; the second study considered cancer incidence in T1D patients [14]. The first study examined 330,000 T2D patients, followed along 2007–2013. Incidence ratios for cancer risk were found increased in both genders for liver, colon, pancreas, kidney, and specifically for prostate in men, and for lung and stomach in women. The second study associated T1D with cancers. Registers from 5 countries were used, in North EU and Australia; in about 9000 patients over 3.9 million people, associations were found, of both direct and reverse sign and in men and women. A proportional hazard model was implemented, revealing for both men and women significance in liver, pancreas, stomach and kidney cancers, for only women significance for ovary and thyroid cancers, for only men significance for colon. A low incidence was revealed for BC and melanoma in women and for prostate cancer in men. Variation was observed across countries, but not in subsites of each of them. The duration of diabetes was also seen to determine an increased incidence at the beginning (first year after diagnosis).

In general (see [15]) T2D presents two features associated with cancer: insulin resistance and hyperinsulinemia. As the association occurs independently on a variable such as BMI, the implication is that T2D might be an independent risk factor for cancer. In particular, breast, colon and pancreas cancers showed positive associations, contrary to prostate cancer. Finally, anti-diabetes drugs can affect cancer cells, and in some cases represent a target for cancer therapies too. As two of the main promoters of cancer in T2D patients are chronic hyperinsulinemia and hyperglycemia, difficulties emerge in some cases. Even if a role is played in the above listed cancers, it is particularly complex the consideration of directionality of association for pancreatic cancer in which diabetes may induce through hyperinsulinemia via enhanced proliferation of islet cells, but also destruction of islet cells and insulin resistance due to cancer may reverse the directionality.

## Systems medicine

### Rationale

A systems approach is particularly effective for deciphering ways through which control can be exerted. As the systems components are considered emergent properties when interactions are observed [16], the underlying mechanism of interest is synergy. Understanding synergy is therefore key. Networks are great inferential instruments for this scope [17]. Clearly enough, not all systems components may be known under some conditions. Then, the problem becomes how this lack of knowledge may affect the functioning of the system. Also, what about the ability to exert control over the system’s dynamics? Instead of looking at individual components, the components ensemble becomes the quantity to look at. An advantage is that the dynamics are likely more easy to interpret and predict. The principle of uncertainty conservation is embedded in such systems [18]; this principle implies the presence of robustness in the systems components due to their features and functions, and with regard to possible perturbing conditions. Ideally, one would seek to regulate the system to have the uncertainty distributed among the least critical components. This way, at least two important effects could be observed: the overall stability would be preserved, and the risk of catastrophic patterns would be reduced.

### Diabetes systems

There are multiple complexities referred to diabetes, onset, progression, trajectories, comorbidities, therapies. Aiming at the ability of exerting control, we can primarily consider the analyses of the effects of therapeutic interventions, which might be seen as constraints imposed to the physiological system. Thus, the ways such constraints are designed obey to the uncertainty conservation principle. Following [18], this means that the systems’ uncertainties due to unknown components (such as genes, proteins, metabolites, ncRNAs, environmental factors etc.) and their functions are re-distributed. Given a reference system, say a diabetes patient, in principle what truly counts is to consider the system’s resilience, say R, and its entropy, say E. These entities are directly correlated, such that dR dE > 0 (with d standing for variation), according to the fluctuation theorem [19]. This is a general feature of cancer systems too, not only diabetes. Cancer is generally observed to be subject to an increase of entropy. This is true more at a local than at a global level, which is in part due to the loss of connectivity effects. Naturally enough, the crucial impact is at the level of design of strategies devoted to drug targets.

### Challenges

The main trends linked to systems medicine applied to diabetes studies are summarized in Table 1.

**Table 1:** Key concepts in diabetes from a systems medicine viewpoint

1. Can diabetes trajectories be considered as multidimensional objects such that associated risks and comorbidities can be more accurately accounted for?
2. Are electronic health records (EHR) innovative or even disruptive when inducing re-phenotyping and new patient stratifications?
3. Can we build effectively actionable clinical decision support systems (CDSS) from predictive modelling and patient-driven analytics?

### Diabetes heterogeneity

Systems medicine implies that inference needs to account for heterogeneous interconnected dynamics. The advantages offered by this multidimensional approach [20] are:

- Empowering pervasive computing, integrating inference models, and leveraging network solutions;
- Optimizing data re-use strategies;
- Validating the significance of evidences by cross–testing and cross-referencing across models and against datasets;
- Generating patient outcomes from large sets of features.

The main operational instrument of large ensemble analysis of data patterns requires that their consistency is established. Finding consistent diabetes patterns helps to manage heterogeneity [21] and associated data complexities [22].

## Precision medicine

**Fig. 1:**
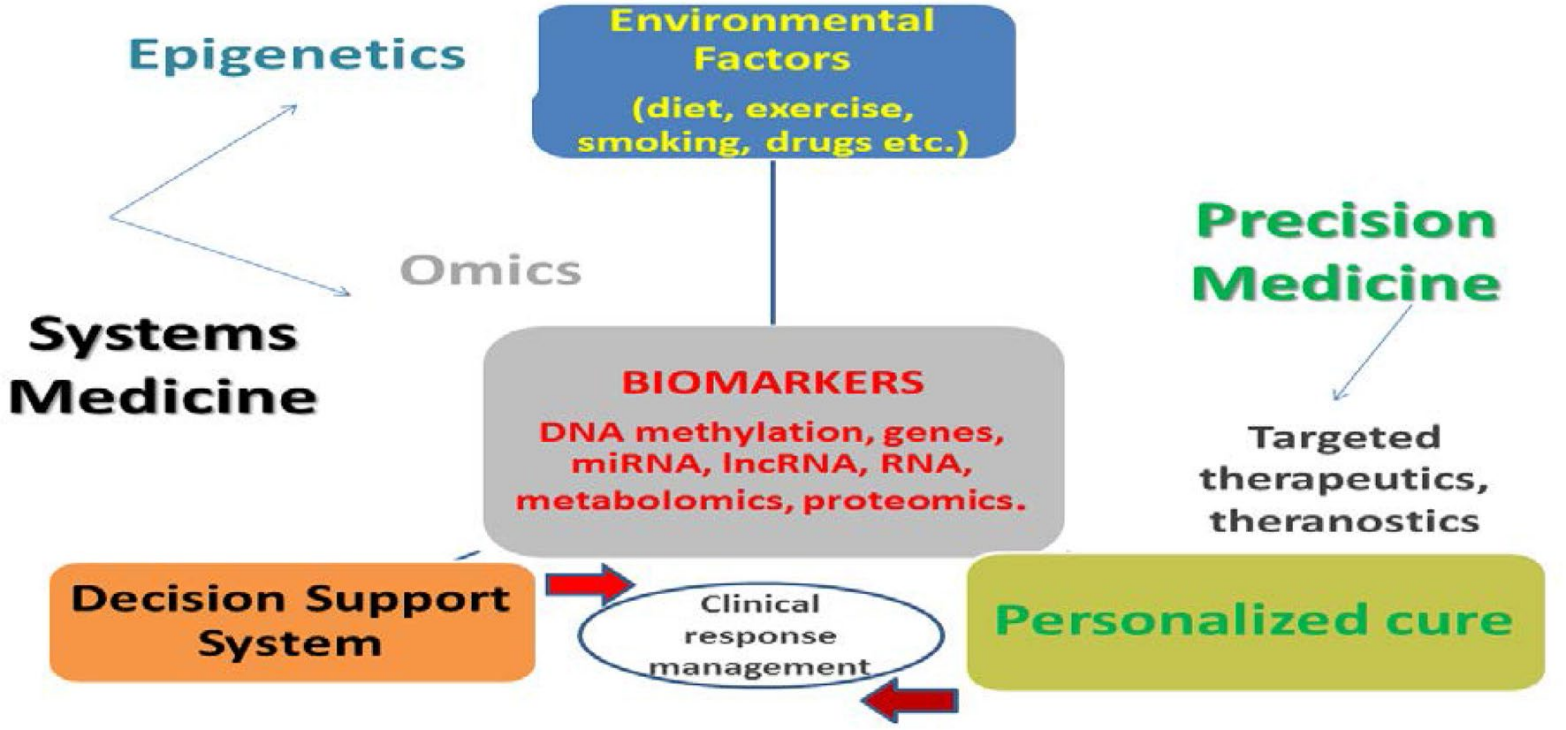
Systems and precision medicine overview

Achieving personalized solutions tailored to each unique combination of patient features is the goal of precision medicine [23–27] (Fig. 1). The variables of interest are those underlying the heterogeneity of patients’ responses to disease progression and treatment. The advantages offered by this approach are:

- Patient-patient similarity networks can represent complex disease patient populations;
- High-dimensional datasets can exploit the topology of re-defined clinical phenotypes;
- Additional genetic and experimental layers can be usefully added to identify specific biomarkers;
- Temporal and spatial aspects can be accounted for.

**Fig. 2:**
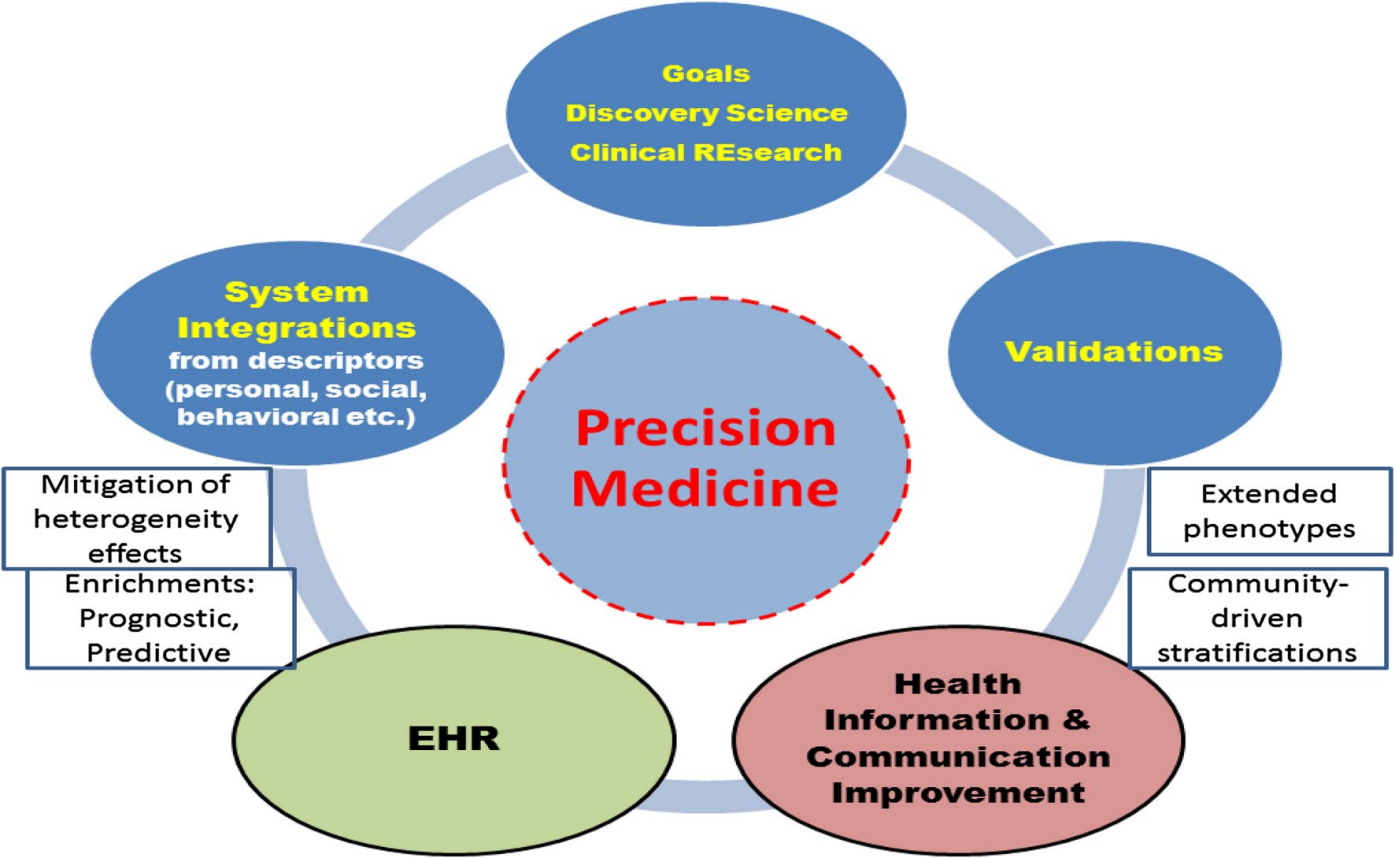
Multidimensionality remains a characteristic also with a precision medicine focus

Precision medicine would require specific accuracy towards components, say drug targets, disease markers, etc., for which not enough information might be individually available unless each of such components are referred within a system, but then becoming subject to increased complexity. This implies a tradeoff between model complexity and model selection. A novel instrument to use is EHR, although they present both strengths and limitations (Fig. 2).

## Diabetes comorbidities and trajectories

### Comorbidities

Comorbidity represents a condition taking place with diabetes too [28]. Both macrovascular complications from heart and cerebrovascular diseases, and microvascular complications manifested as nephropathy, neuropathy and retinopathy are known to occur at various ages [29, 30]. Tissue disruption in pancreatic beta islets can depend on lymphocyte infiltration, inflammation and immunological conditions. Strong epigenetic influences have also been described, and in particular histone modifications have revealed associations with hyperglycemia and pro-inflammatory phenotypic relevance for diabetic complications. Notably, the nutritional and environmental factors linking diabetes, obesity and cardio-metabolic diseases make the behavioral aspect an important basis of the pharmacological therapy [31]. Many data are currently transferred into record of the above factors and influences. Learning in EHRs is therefore a process dependent on how precisely the algorithms to be used may adapt to the variety of data. This process affects also the way we can infer on comorbidities. These are observed linked disease patterns at population scale, but their trajectories can become highly relevant when diabetes onset and the age at the time of onset are considered. This is especially important because of the incorporation of probabilities of early events in development, which are causal to the insurgence of diseases.

The computational goal is to embrace the multidimensionality of the problem by a variety of inference approaches, considering the constraints needed for the multidimensional factors to build maximally informative stratifications. In particular, diseases like diabetes show metabolic links that exert impacts for patient stratifications. What is currently lacking is an assessment of significance of comorbidity relationships across the dimensions and through independently validated datasets. Significance and validation are therefore two main criteria for choosing statistical inference approaches heading to (1) novel stratification of patients based on complex risk profiles; (2) digital biomarkers for disease progression and response to therapy; (3) new therapeutic strategies for composite targets.

### Trajectories

Building trajectories implies the need of considering the dynamic aspect of all dimensions. Attractor states can be present. Attractors are stable points to which the system would return after small shocks [32]. Dynamically assessed maps would be very informative in unconstrained systems, despite the uncertainty related to causality remains. Networks would probably deliver useful modular configurations, but should be tuned especially to sense early warnings, thus predicting the occurrence of perturbations able to induce critical transition phases, and also causing network re-modulation [17].

A complex disease signature can be computationally built from predictive measurements of clinical outcomes, such as disease state, survival time, response to therapy, including a set of genetic and biomolecular features identifying clinical phenotypes. In particular, aggregating such features produces effects at systems scale. This means that the prediction power holds for phenotypes of clinical interest in the observed patient (diagnostic, i.e. progression of comorbidity; therapeutic, i.e. response to therapy and toxicity; prognostic, i.e. time to recurrence or death), and in the unseen patient (risk profile, prediction of disease occurrence). When such features are considered at network scale, one of the advantages is that modularity can identify a number of functionally active, connected and cohesive groups of features. This improves the power for detecting perturbations during the onset and the progression of comorbidity, and allows for better interpretability of the dynamics at different time spans.

Recently, a study has proposed strategies for building diabetes trajectories and associated risks [33]. A practical definition of trajectory was indicated as follows: a sequence of conditions that are crossed before getting to T2D, and during which some comorbidities are presented (hyperlipidemia, hypertension, impaired fasting glucose). Predictive models can serve the scope of finding atypical trajectories associated with risks of diabetes. Data are taken from EHRs (Rochester, Epidemiology Project linked to Mayo Clinic, 13-year data). The results confirm one significant trajectory for two-thirds of patients, HLD-HTN-IFG-T2D, while other minor ones distribute across the remaining one-third of patients. Limitations were reported, according to the timing of diagnoses (pre-existing?), missing or false positives, but also slow-onset comorbidity conditions inducing to focus on patient with fast progression. Phenotyping algorithms that are selected are also a limiting factor, contributing to analysis bias. Other studies [34, 35] have proposed diabetes trajectories built from clustering of diagnoses satisfying minimal numerical requirements to establish trajectory directionality. Notably, from EHR data collected for 15 years on 6.2 million. Danish patients, more than 1100 trajectories were built to demonstrate that retinal disorders were systems markers of diabetes vasculopathy and especially renal failure worsened for the presence of vitreous body disorders. The same group of authors in a similar study from EHR data (1996–2014) on 6.6 million patients (120,000 suffering from sepsis), found recurrent significant mortality trajectories that included non-insulin dependent diabetes. Among the multiple routes from diabetes to sepsis, three main ones were identified through ulcers, pneumonia, anemia, and more that 2200 trajectories were found with at least four diseases involving more than 20 patients.

## Diabetes heterogeneity via re-phenotyping

Use of knowledge bank approaches implies that estimated risk factors are reclassified over patients (inter-patient risk reclassification) [36, 37]. At the individual level another aspect that is worth considering is the risk estimate as the result of an aggregate of information on multiple disease features. The question then is: how EHR are expected to improve diabetes care? Among the indicated benefits the following reported in Table 2 are described.

**Table 2:** Benefits from EHR

a. Generate and use of patient lists for scopes of research and health care quality improvement
b. Set alert systems with warnings and reminders on preventive care and screenings
c. Improve doctor-patient communications and promote use of patient reports
d. Enhance clinical decision making by better monitoring of patient history trends
e. Improve management of prescriptions

More in general, EHR advantages and limitations may be listed as in Table 3.

**Table 3:** Pros and Cons of EHR

Advantages
EHR synergies inducing deep phenotyping and marker re-modulation
Clinical decision support systems (CDSS) providing valuable deliverables
Network community-driven stratifications as inference drivers
Limitations
Geo-differentiation creates heterogeneity and needs protocols for effective aggregation of patients’ information
Records distillation for curating information towards decisions, still facing incompleteness
Embedding of CDSS for standardization and actionable decision making

In summary, two main aspects are worth future consideration:

1. Electronic health systems represent complex junctions of phenotypes: The search for consistent patterns across patients in different data collections will be central to the re-definition of diabetes features.
2. Repurposing phenotypes will be central to the future developments in precision medicine: Personalized solutions will consider distinct patients’ features and many differential therapeutic paths will be designed.

### Impact of database heterogeneity

Another important question refers to the fact that with alternative database resources that merge into EHRs, what is the expected effects on the quality of results? Interesting evidences have appeared showing that the same drug was reported to yield conflicting significant results (decreased vs increased risk) [38]. However, a possible underestimation of heterogeneity may underlie such uncertainty. Simply pooling data or performing meta-analysis is not a best practice towards good outcomes, in particular toward causal interpretation. With large heterogeneity, what is the ability of observational data to address clinical questions? We need joint quantitative and qualitative (study-specific) evaluations. It is important to notice that EHRs originate from diverse data capture processes, mostly separated from research intentions (claims reflect reimbursement process, thus diagnosis codes). EHRs are often conceived to support clinical care, through information required by providers to perform services not medical histories. Different source populations, geographic, demographic, disease severity, longitudinal lengths of studies, temporal quality aspects are determinants inherently diversified.

### Patient stratifications

The clinical and genetic complexity of T2D patients’ heterogeneity present opportunities to refine the current symptom-based subtypes. Compiling genetic, molecular and clinical features of patients, these can be clustered into subgroups allowing more informed decisions about treatments. The previously mentioned knowledge bank approaches are implemented to form clinically relevant clusters, whose size depends on the variables that are used. Compared to previous knowledge bases (built from expertise, judgment etc.) the new ones work under different metrics that need to assemble superior data diversity (i.e. newly revealed layers of disease heterogeneity), and thus face new complexities (increased number of predictors, risk factors and confounders to account for). The goal is to yield accurate risk profiling assuming proper sample size has been reached in the knowledge bank.

Consequently, multilevel models are the expected analytical solutions to be derived from knowledge banks. This in light of the ultimate goal of precision medicine: to inform patients about optimal treatment choices. When a knowledge bank approach from EHR is followed, a natural question is to assess what fluctuations can be measured with reference to treatment decisions operated on the basis of the bank versus other options excluding the bank source. The more the predictors contributing to risk estimates are different in the two scenarios, the more we can expect increased variability computed around an hypothetical global average taken across the two options which is induced by the values computed in the former one. This translates into the evidence of a significant differential therapy path for a substantial number of patients. An entropy increase is expected from the combination of sources compared to individual ones, which makes the control of delta resilience R crucial. The latter entity is going to depend necessarily on sample size, and at a global scale we can say that the more samples are available and the more a knowledge bank can be representative of reference populations. Therefore, with increasing sample sizes, our chances of finding dR > 0 are increasing too. When we reason in terms of stratification, the uncertainty conservation principle has necessarily lesser chances to be verified, due to groups of different sizes and entropies, surely resilient due to their specific homogeneity. In such scenario, it becomes likely more relevant to explore possible synergies, and design interconnections through networks.

Going back to diabetes genomics, some aspects are relevant to knowledge bank approaches. Apart from the limited power of detecting associations, biobank studies aim to link genetic information to EHR phenotypes, despite the limitations from non-standard lab tests, ambiguity of diagnosis codes, geolocations, database diversity. A recent study [39] has identified three T2D subtypes on the basis of comorbidities (more than 11,000 patients). Genomics can extend the stratification ability to cover genetically identified treatment strata. EHRs can instead enrich strata through identification of geographic areas with differential diabetes treatment (at risk neighbourhood-level community-based interventions) and from geo-localized environmental factors (say for instance triggers of autoimmunity), thus improving the precision prevention plan. Another study on more than 11,000 T2D patients analyzed patient similarity with a topological approach and identified 3 clusters with specific features, confirmed then by use of SNPs [40]. Conversely, Big Data will require additional multiple testing penalties, need for replication, even if reproducibility of results may be an issue and interpretability too.

### Diabetes data fusion strategies

Data fusion stands for the analysis of many datasets such that relevant information can be extracted from their interaction. The main issue is that datasets can be generated by different or independent experiments, and by pooling them together it might be not immediately clear how the possible interactions can be established. The data pool can present multimodality, and to some extent some underlying data relationships might be revealed when such data are fused. The synergy principle applies as the ensemble of datasets is more than the simple sum of them. In other terms, the data space includes some links between data that allow synergy to be exploited, thus creating a feature of diversity otherwise unseen from the separate datasets. We need a sufficient number of constraints to determine this kind of problems. However, any constraint represents also a diversity factor to exploit, thus useful information toward the identifiability of the problem. Dataset linkages are precisely the kind of diversity we aim to find when fusion is operated.

Some challenges remain as open problems summarized in Table 4.

**Table 4:** Data fusion critical aspects

Balancing information from different sources or origins
Managing conflicting, contradicting and inconsistent data
Handling missing values
Differentiating between hard and soft data links, i.e. considering the random processes from which the data are generated as subject to the same parameters, or instead accounting just for covariations, dependencies, similarity/dissimilarity etc.
Establishing loss or objective functions and regularization/penalty terms

In other terms, the main problem becomes one of how to design structured data fusion strategies allowing the embedding of data linkages and synergies useful to optimize inference about diabetes.

## Added value from recent studies

How T2D screening can find benefit in EHRs? Assumption is that the phenotyping in EHRs, despite incomplete, heterogeneous and not systematic, can still add value to the more conventional models. In a recent study [41], 9948 US patients formed the reference for building a pre-screening tool to predict T2D using two main methods, multivariate logistic regression and random forest model. Three settings were considered: (a) a full EHR model including prescribed medications, diagnoses and conventional predictors; (b) a restricted EHR model with no medication records; (c) a conventional model with basic predictors and interactions (BMI, age, sex, smoking, hypertension). Using (a) or (b) got better results than (c), in terms of significance. Adding EHR phenotypes improved classification. Some surprise factors associated with diabetes. Contradicting results came out for side effects of prednisone, migraine, allergies, or even smoking, negatively correlated with T2D, while enalapril, various infections, bronchitis revealed positively correlated.

Limiting factors were identified, such as incomplete family history, missing risk factors, such as ethnicity and socioeconomic status. Neither prognostic nor causal factors were included. While risk scores could be computed, and can be further refined to a general patient profile, also the medication classes could be better assessed in T2D context. The main criticism is that results are usually more or less consistent with the knowledge we have from maybe other models built on different premises. Models to some extent drive the consistency of results, therefore present a margin of error which remains difficult to quantify. Use of ensemble data and models could attenuate this risk. Confidence measures could be better supported this way. Otherwise, shift of paradigm can be priority. Instead of assessing standalone model predictions, real-time tracking of their trajectories could reveal patterns consistent in time and space, especially in light of the diversity of data in EHRs.

In another study [42] the problem of defining phenotypes was addressed, as multiple ways of assembling their components (frequency, clinical context, time factor among other) may change phenotypic cross-performance. Thus, 8 phenotypes were scrutinized, of which 2 were ICD-9 codes, one was hemoglobin A1c, another diabetes medication, diagnoses codes, lab results, medications, secondary data (demographic, social, environment, census, etc.) to identify patients, health management for public health intervention and epidemiology, EHRs and genomics, and combinations. The target population was made of 173,503 adults well localized in US (NC) meeting the definitions. Sensitivity and specificity in identifying T2d were quite variable across the phenotypes. 45% of patients identified with diabetes had not diabetes (from clinical expert chart review). Hard to separate T1D and T2D (say, obese patients in insulin therapy).

## EHR-guided precision medicine

### Impacts

Strong impacts can be derived from precision medicine and EHR with regard to our understanding of complex diseases and management of comorbidities affecting an increasing amount of people. Precision medicine relevance is destined to grow with the development of EHR. One priority is to find synergies between phenotypes and markers of diseases and comorbidities. The expected deliverables include better support to personalized decisions and more accurately stratified risk profiles.

An effective interoperability is required between a few elements: data re-use; predictive models; algorithm efficiency; disease ontologies; multiplexed networks. A critical assessment of the integration of Precision Medicine within the EHR framework is needed in light of evidences, studies and experiences of leading experts in the fields and their newest research work.

Precision Medicine somehow represents an arrival point. The previous era of P4 has established a shift of paradigm in the medical field. Great progresses were observed in both experimental biological and clinical levels. In genetics, a wealth of applications of Next Generation Sequencing techniques has determined a revision of old principles and allowed new projects (see ENCODE, https://www.genome.gov/10005107/encode-project/; Roadmap Epigenomics, http://www.roadmapepigenomics.org/; 1000 Genomes, http://www.internationalgenome.org/, etc.).

In turn, a multitude of new evidences that were generated, surely are contributing to determine the centrality of EHR. Both advantages and open problems appear ad the horizon. Among the advantages, data synergies leading to the integration of heterogeneous sources of information, the definition of deep phenotyping and markers re-modulation; the establishment of clinical decision support systems. Among the limitations, currently some problems remain to be solved, involving geo-differentiation and ethnic balance, protocols for sharing of digital information, interoperability between different record types (structured and non) to optimize the process of decision making in an actionable way.

Precision medicine is destined to shape the future of medical research, and in a measure dependent on EHR’ contributions that is still difficult to estimate at the present time. The interest and debate on Precision Medicine and EHR, are both escalating. Some of the emerging aspects are critical. The link between “precision” and “big data’ conducts the reasoning over the sufficiency of information in EHRs as the really necessary point from which to start a combined analysis of the two topics. The medical field is the gravity point of the interconnected fields. It is also perfect time for promoting the relevance of translational medicine, for instance through the synergies between biomedicine and quantitative/computational disciplines contributing to the developments in EHR analysis, modelling and comorbidity inference.

In Precision Medicine, the importance of each patient will be valuable as much as the centrality of any type of subpopulation study. Medical researchers and physicians would agree over the importance of supporting their acquired scientific and clinical knowledge with information from a variety of sources, both theoretically and practically inspired. Ultimately, by looking at timely, organized, standardized and accurate data, exactly the opportunity that EHR present, the quality of care can only benefit. Reasoning of strengths and weaknesses inherent to all such sources is therefore crucial to determining how the future medical protocols will be established. The process of democratization enabled by EHR is far from being achieved at this time when we write, but planned to be so at some point. The naturally expected consequence is improved quality of care. Despite the information and communication and exchange standards of efficiency and interoperability that are aimed to be reached by EHR are still matter of debate, it is commonly recognized a new role of patients in this overall process, in particular an active one primarily due to the relevance of factors that are not under the direct control of doctors (life-style, technology and social factors). Patients will surely profit from a better understanding of the role that Precision Medicine may have in light of the possible synergies and limitations of the medical data frames, thus expanding their knowledge base.

### Claims data and risk factors

Risk prediction model for T2D was proposed, finding increased power from surrogates of variables that are usually missed. Then, the relative importance of such factors in part depends on how early they may predict T2D onset. In a recent study [43], a set of 42,000 variables was built for explaining individual medical states (4.1 million patients, from an insurance program within 2005–2013, mostly in Philadelphia US). Some limitations have been emphasized. One of the problems is the detection of contextual anomalies, i.e. data instances appearing in contexts contrary to expectation. This implies that we should know the distribution of patients expected responses and obtain the deviations allowing for detection of anomalies. Defining outlying patterns requires establishing distance metrics in which to assess features and identify singleton patients. For model purposes, these latter should be eliminated, but probably they represent important anomalies with reference to treatment medications or related comorbidities. Some sparsely represented features can be associated with anomalies, therefore. Also, some confounding factors may become more measurable from EHR.

## Conclusion

The path forward with *Big Data*, not only the likely increase of dimensionality might augment the general complexity (spurious correlations, error propagation etc.) and affect the confidence in models, but in many cases the original data are unstructured or based on a huge number of primitives, and in both cases either transformations or reductions are pursued. In general, the patterns at individual and population levels may differ substantially, and thus be hardly summarized by some statistics or predicted with some confidence. It is expected that with the integration of information from a variety of sources, the assimilation of the whole data spectrum could not incur in significant loss of information (a good example might be again a subset of patients responding to the same treatment). Therefore, Big Data in medicine, for instance, would benefit from the ability to recognize disease heterogeneity and to stratify even further in order to be more accurate in the assessment of therapies. In such regards, we might thus consider the ‘blessing of *Big Data*’.
